# Reduzierung des Zuckerkonsums für eine bessere Mundgesundheit – Welche Strategien sind Erfolg versprechend?

**DOI:** 10.1007/s00103-021-03349-2

**Published:** 2021-05-20

**Authors:** Anja Heilmann, Sebastian Ziller

**Affiliations:** 1 Department of Epidemiology and Public Health, University College London, WC1E 6BT London, Großbritannien; 2 Abteilung Prävention und Gesundheitsförderung, Bundeszahnärztekammer (BZÄK), Berlin, Deutschland

**Keywords:** Zucker, Mundgesundheit, Karies, Kommerzielle Gesundheitsdeterminanten, Gesundheitsstrategien, Sugar consumption, Oral health, Dental caries, Commercial determinants of health, Public health strategies

## Abstract

Strategien zur Gesundheitsförderung können einen wesentlichen Beitrag leisten, um nichtübertragbare chronische Krankheiten zu verhindern. Die wesentlichen nichtübertragbaren Erkrankungen in der Zahnmedizin sind Zahnkaries und Parodontalerkrankungen. Zucker spielt sowohl bei der Entstehung von Zahnkaries als auch von Übergewicht und dessen Folgen für die Allgemeingesundheit eine ursächliche Rolle und ist daher als wichtige kommerzielle Gesundheitsdeterminante mehr und mehr ins Blickfeld von Wissenschaft und Gesundheitspolitik gerückt. Existierende Strategien zur Reduktion des Zuckerkonsums zielen jedoch häufig auf Maßnahmen zur individuellen Verhaltensänderung ab und lassen dabei die Rolle von gesellschaftlichen und kommerziellen Einflüssen außer Acht.

In diesem Artikel beschreiben wir die aktuellen Empfehlungen der Weltgesundheitsorganisation (WHO) zum Zuckerkonsum, Daten zum Zuckerverzehr in Deutschland sowie die sozialen und kommerziellen Faktoren, welche den Zuckerkonsum beeinflussen. Grundlegende Prinzipien der Gesundheitsförderung werden dargelegt und sich daraus ergebende Strategien zur Zuckerreduzierung diskutiert. Dabei werden konkrete Beispiele für Upstream- und Downstream-Ansätze benannt und Möglichkeiten der Einflussnahme durch die zahnmedizinische Community in Politik und Praxis aufgezeigt.

## Abstract (en)

Health promotion strategies can make a significant contribution to the prevention of non-communicable diseases. The most important non-communicable diseases affecting the mouth and teeth are dental caries and periodontal disease. Sugar is a causal factor in the development of dental caries, obesity and obesity-related diseases. Sugar is therefore recognised as an important commercial determinant of health and a public health priority. However, existing strategies to reduce sugar consumption often focus on individual behaviour change while ignoring the role of the wider social and commercial environment.

In this article we describe the current recommendations on sugar intake made by the World Health Organization, existing data on sugar intake in Germany as well as the social and commercial factors that influence sugar consumption. Important public health principles will be outlined, followed by a discussion of appropriate sugar reduction strategies. Examples for upstream and downstream approaches to prevention are presented and opportunities for dental health professionals to influence policy and practice highlighted.

## Einleitung

Im Januar 2021 verabschiedete der Exekutivrat (EB148) der Weltgesundheitsorganisation (WHO) mit breiter Unterstützung eine Resolution zur Mundgesundheit – laut WHO-Generalsekretär Dr. Tedros in der Geschichte der Mundgesundheit ein wegweisender Meilenstein. Nachdem die Mundgesundheit über Jahrzehnte hinweg nur eine geringe gesundheitspolitische Priorität genoss, spiegelt die aktuelle Resolution ein Umdenken auf höchster Ebene wider, das die Mundgesundheit als einen wesentlichen und untrennbaren Bestandteil der Allgemeingesundheit anerkennt [1]. Die Zahlen sprechen für sich: Unbehandelte Karies der bleibenden Zähne ist die weltweit häufigste aller chronischen, nichtübertragbaren Erkrankungen. Im Jahr 2015 wurde die Zahl der weltweit Betroffenen auf 2,5 Mrd. und die altersstandardisierte Prävalenz auf etwa 34 % geschätzt [2]. Zudem gibt es bei der Kariesverteilung in der Bevölkerung in allen Altersgruppen ein starkes soziales Gefälle [3]. Auch in Deutschland ist Karies weitverbreitet. Während laut Fünfter Deutscher Mundgesundheitsstudie (DMS V) die Kariesprävalenz bei Kindern und Jugendlichen über die vergangenen Jahrzehnte erfreulicherweise stark zurückgegangen ist und 81 % der 12-Jährigen 2015 kariesfrei waren, wurde bei knapp 25 % der jungen Erwachsenen sanierungsbedürftige Karies festgestellt und weniger als 3 % hatten keine Karieserfahrung [4].

Karies ist keine harmlose Erkrankung – sie beeinträchtigt Gebissfunktion und Ästhetik und kann die Lebensqualität erheblich mindern. Karies verursacht Schmerzen und kann vor allem bei Kindern zu Appetitverlust, Schlaflosigkeit, Konzentrationsstörungen sowie Schulabwesenheit und sogar Gewichtsverlust führen [5]. Karies ist neben der Parodontitis eine der Hauptursachen für Zahnverlust und ihre Behandlung ist zeitaufwendig und teuer.

Karies ist jedoch überwiegend vermeidbar. Die entscheidende Ursache für die Entstehung von Karies ist übermäßiger Zuckerkonsum [6, 7]. Es ist wichtig, diese Tatsache klar hervorzuheben. Faktoren wie Speichelfluss und Aufnahme von Fluoriden wirken dem Kariesprozess entgegen und spielen somit eine wichtige Rolle, werden aber als Effektmodifikatoren angesehen [6]. Der Zuckerkonsum selbst wird wiederum durch umweltbedingte und psychosoziale Faktoren bestimmt [8].

Ein hoher Zuckerkonsum ist auch mit einem erhöhten Risiko für Parodontalerkrankungen assoziiert. Es wird angenommen, dass durch die Nahrung aufgenommener Zucker chronisch entzündliche Erkrankungen wie Parodontitis begünstigt [9].

Die wirtschaftlichen Kosten für die Behandlung von Karies, Parodontitis und Zahnverlust sind immens. Laut einer Modellrechnung beliefen sich die direkten und indirekten Kosten von durch übermäßigen Zuckerkonsum verursachten Zahnerkrankungen im Jahr 2010 auf global 172 Mrd. US-Dollar. Für Deutschland wurden dabei pro Jahr und Person 210 € an Zahnbehandlungskosten errechnet [10]. Angesichts der erheblichen Beeinträchtigungen der Lebensqualität für den Einzelnen sowie der hohen Kosten für Gesundheitssystem und Gesellschaft stellen diese zuckerbedingten Erkrankungen also ein signifikantes Gesundheitsproblem dar.

Zucker spielt aber nicht nur für die Mundgesundheit eine Schlüsselrolle. Ein hoher Zuckerkonsum, vor allem durch zuckrige Getränke, geht mit einem erhöhten Risiko für die Entstehung von Übergewicht und Adipositas sowie deren Folgeerkrankungen einher [11]. Wissenschaftlich belegt sind außerdem Zusammenhänge zwischen Zuckerkonsum und Diabetes Typ 2 [12] sowie Herz-Kreislauf-Erkrankungen [13]. Als Risikofaktor lange vernachlässigt, ist Zucker infolge dieser Erkenntnisse mehr und mehr ins Blickfeld der Gesundheitspolitik gerückt.

In diesem Beitrag beschreiben wir die derzeit geltenden Empfehlungen der WHO zum Zuckerkonsum sowie Daten zum Verzehr in Deutschland. Unser Ziel ist es, wichtige Faktoren, die den Zuckerkonsum beeinflussen, zu beleuchten und Konzepte und Strategien zur Zuckerreduzierung darzulegen, um die Mundgesundheit zu verbessern.

## WHO-Richtlinie zum Zuckerkonsum

**Figure 1:**
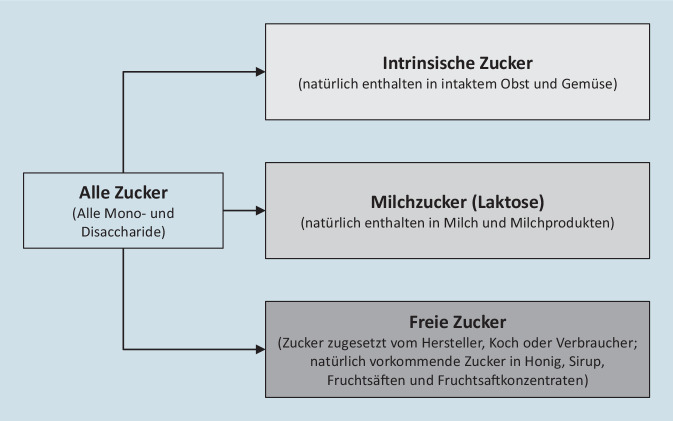

Im Jahr 2015 wurde von der WHO eine evidenzbasierte Richtlinie zum Zuckerkonsum veröffentlicht [14]. Die WHO unterscheidet zwischen intrinsischem Zucker, der in frischem, intaktem Obst und Gemüse vorkommt, Milchzucker (Laktose), der natürlich in Milch und Milchproduckten enthalten ist, und dem sogenannten freien Zucker (Abb. 1). Die WHO-Richtlinie bezieht sich ausschließlich auf freie Zucker. Die Definition freier Zucker umfasst alle Mono- und Disaccharide, die Lebensmitteln und Getränken vom Hersteller, Koch oder Verbraucher hinzugefügt werden, sowie die natürlich vorkommenden Zucker in Honig, Sirup, Fruchtsäften und Fruchtsaftkonzentraten [14]. Im Gegensatz zu den freien Zuckern gelten sowohl Milchzucker als auch die in frischem Obst und Gemüse^1^ enthaltenen Zucker als gesundheitlich unbedenklich [14].

Die WHO-Richtlinie basiert hauptsächlich auf 2 systematischen Reviews, welche den Stand der epidemiologischen Forschung zur Bedeutung freier Zucker sowohl für ungesunde Gewichtszunahme [11] als auch Zahnkaries [7] zusammenfassen.

Um das Risiko von Übergewicht und dessen Folgeerkrankungen sowie von Karies zu reduzieren, empfiehlt die WHO, die Aufnahme freier Zucker in allen Lebensphasen auf unter 10 Energieprozent zu senken (nachdrückliche Empfehlung). Bei einer Aufnahme von 2000 Kilokalorien pro Tag entspricht dies rund 50 g Zucker (ca. 10 Teelöffel) für einen durchschnittlichen Erwachsenen. Zusätzlich hält die WHO eine weitere Reduktion auf unter 5 Energieprozent (also täglich nicht mehr als 5 Teelöffel bzw. 25 g Zucker für Erwachsene) für sinnvoll (ergänzende Empfehlung; [14]).^2^

Die WHO-Empfehlungen stellen für nationale, gesundheitspolitische Entscheidungsträger einen Referenzrahmen dar und sollen eine treibende Kraft für gesundheitspolitische Veränderungen in Richtung Zuckerreduktion sein [15]. In Deutschland haben die Deutsche Adipositas-Gesellschaft e. V., die Deutsche Diabetes Gesellschaft e. V. und die Deutsche Gesellschaft für Ernährung e. V. ein Konsenspapier veröffentlicht, in dem sie sich der nachdrücklichen WHO-Empfehlung für eine maximale Zufuhr freier Zucker von weniger als 10 % der Gesamtenergiezufuhr anschließen [16].

## Zuckerkonsum in Deutschland

Zu den wichtigsten Quellen freier Zucker in der Ernährung der Deutschen gehören zuckerhaltige Getränke, Süßigkeiten und Backwaren [17]. Für Kinder spielen Frühstückszerealien mit hohem Zuckergehalt eine wichtige Rolle [18].

Repräsentative Daten zum Zuckerkonsum der erwachsenen deutschen Bevölkerung wurden zuletzt durch die zwischen 2005 und 2007 durchgeführte Nationale Verzehrsstudie II erfasst. Demnach lag der durchschnittliche Verzehr freier Zucker für Männer bei täglich 78 g oder 13 Energieprozent und für Frauen bei 61 g oder 14 Energieprozent. Der Zuckerkonsum ist jedoch stark altersabhängig. Bei den Männern war er am höchsten bei den 15- bis 24-Jährigen mit durchschnittlich 17 Energieprozent pro Tag und am niedrigsten bei den 65- bis 80-Jährigen mit etwa 10 Energieprozent pro Tag. Bei den Frauen lagen die entsprechenden Werte bei 18 % und 12 % [17]. Vor allem für junge Erwachsene sind diese Werte weitaus höher als das von der WHO empfohlene Maximum von 10 oder gar 5 Energieprozent.

Die Messung des Konsums freier Zucker bei Kindern (3–18 Jahre) in der Studie DONALD (Dortmund Nutritional and Anthropometric Longitudinally Designed) ergab für den Zeitraum zwischen 2005 und 2016 einen Wert von durchschnittlich 16–17 Energieprozent für Mädchen und Jungen. Laut DONALD war der Verzehr bei jüngeren Kindern höher als bei älteren. Zwischen 2010 und 2016 verzeichnete die Studie einen erfreulichen Abwärtstrend, allerdings blieb der Zuckerkonsum nach wie vor deutlich über der von der WHO empfohlenen Menge [19].

**Figure 2:**
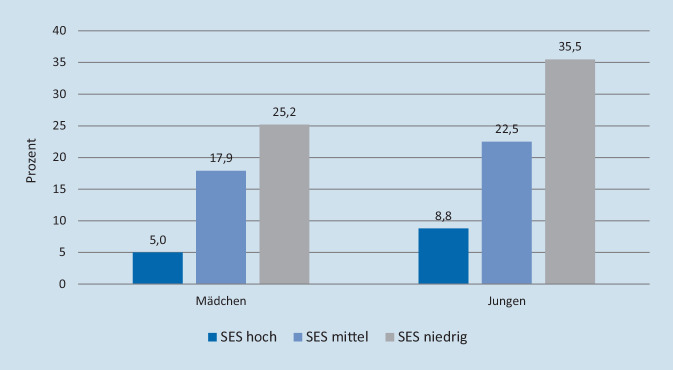

Es gibt deutliche Hinweise, dass der Zuckerkonsum bei Kindern und Jugendlichen sozial ungleich verteilt ist. Abb. 2 zeigt Daten der Studie zur Gesundheit von Kindern und Jugendlichen in Deutschland (KiGGS) Welle 2 (2014–2017) für den täglichen Konsum zuckerhaltiger Erfrischungsgetränke (z. B. Cola, Limonade; Fruchtsäfte nicht eingeschlossen). Die Daten dokumentieren ein klares soziales Gefälle: Ein täglicher Konsum wurde weit häufiger für Kinder aus Familien mit niedrigem sozioökonomischen Status (SES) angegeben als für Gleichaltrige aus Familien mit höherem SES [20].

## Wodurch wird der Zuckerkonsum beeinflusst?

### Evolution und globaler Ernährungswandel

Menschen sind durch die Evolution auf eine Vorliebe für süße, fetthaltige, energiereiche Nahrung programmiert – für unsere steinzeitlichen Vorfahren brachten diese Präferenzen einen klaren Überlebensvorteil. Durch den globalen Ernährungswandel leben wir heute allerdings in einer Welt, in der zuckrige und hochkalorische Nahrungsmittel jederzeit billig verfügbar sind und auch stark beworben werden. Dieses Missverhältnis zwischen inhärenten Vorlieben und einer modernen Umwelt, in der diese Vorlieben für Krankheiten anfällig machen, ist ein wesentlicher Grund für die weltweit starke Zunahme chronischer Erkrankungen über die vergangenen Jahrzehnte [21]. Es erklärt auch den nur mäßigen Erfolg von Maßnahmen, die allein auf individuelle Verhaltensänderung durch Gesundheitserziehung abzielen, aber Lebens- und Konsumwelten unangetastet lassen [22, 23].

### Soziale Gesundheitsdeterminanten

Wenn es um Gesundheitsverhalten geht, wird häufig von „Lebensstil“ (Lifestyle) gesprochen, als wären dies Entscheidungen, die unabhängig von den Lebensumständen in einem „Vakuum“ getroffen werden [8, 24]. Dabei wird oft übersehen, dass nicht alle Menschen über die gleichen Ressourcen und Entscheidungsmöglichkeiten verfügen. Menschliches Verhalten wird von unzähligen Faktoren geprägt, von denen viele außerhalb der Kontrolle des Einzelnen liegen [25]. Diese Faktoren werden unter dem Begriff der *sozialen Gesundheitsdeterminanten* zusammengefasst und erklären die in fast allen Gesellschaften anzutreffenden sozialen Ungleichheiten bei der Verteilung von Krankheit und Gesundheit [26]. Auch der Zuckerkonsum wird von einem komplexen Zusammenspiel sich gegenseitig beeinflussender Gegebenheiten bestimmt [8]. Auf oberster Ebene gehören dazu z. B. die wirtschaftlichen Rahmenbedingungen, die für Produktion, Zusammensetzung, Handel, Verfügbarkeit und Erschwinglichkeit von zuckerhaltigen Produkten entscheidend sind. Ebenso wichtig sind die Marketingstrategien der Lebensmittelindustrie sowie der Umfang, zu dem diese vom Staat reguliert werden [27]. Das Angebot von Speisen und Getränken in Lebenswelten wie Schulen und Arbeitsplätzen bestimmt maßgeblich, was dort konsumiert wird, und kann zu sozialen Normen beitragen. Auf individueller Ebene spielen Einkaufsmöglichkeiten im Wohngebiet und verfügbares Einkommen eine Rolle – so sind (bei gleicher Menge an Kalorien) frisches Obst und Gemüse vergleichsweise teurer als zuckrige und fettreiche Produkte [28]. Ebenfalls wichtig sind die Erwartungen von Familie und Freunden, aber auch ernährungsbezogenes Wissen sowie die Fähigkeit und Möglichkeit, gesundes Essen selbst zu kochen. Nicht zu vernachlässigen sind psychosoziale Faktoren – für Menschen, die unter viel Stress stehen, kann süßes Essen ein Bewältigungsmechanismus sein. Außerdem besteht ein Zusammenhang zwischen depressiver Symptomatik und Essverhalten [29].

### Zucker als kommerzielle Gesundheitsdeterminante

Das Konzept der *kommerziellen Gesundheitsdeterminanten* wurde 2016 von Kickbusch und Kollegen formuliert. Gemeint sind damit jene Strategien und Vorgehensweisen der privaten Industrie, welche den Konsum gesundheitsschädlicher Produkte vorantreiben [30].

Die wirtschaftlich mächtige, politisch einflussreiche, globale Zuckerindustrie ist ein Paradebeispiel für kommerziellen Einfluss mit negativen Folgen für die Gesundheit der Bevölkerung. Zucker ist billig und die Gewinnspannen sind hoch. Die Strategien der Industrie ähneln denen der Hersteller anderer gesundheitsschädlicher Produkte, wie Tabak und Alkohol, und sind in den letzten Jahren mehrfach beschrieben worden [31, 32]. Werbung und aggressives Marketing machen einen bedeutenden Teil der Vermarktungsstrategien aus [33]. Fast jeder kennt die eine oder andere Darstellung der Ernährungspyramide, wobei fettreiche und süße, als ungesund geltende Nahrungsmittel in der Spitze der Pyramide dargestellt werden, um zu vermitteln, dass sie nur einen sehr geringen Anteil der Nahrung ausmachen sollten. Würde man jedoch eine Pyramide aller Nahrungsmittel zeichnen, wie sie anteilig in der Werbung vorkommen, würden dieselben ungesunden Produkte fast die gesamte Pyramide einnehmen [34]. Besonders problematisch ist die gezielte Vermarktung stark zuckerhaltiger Produkte, die sich direkt an Kinder richtet. Laut einer Untersuchung des Max Rubner-Instituts weisen z. B. speziell als Produkte für Kinder vermarktete Frühstückszerealien und Milchprodukte häufig einen besonders hohen Zuckergehalt auf [18].

Weniger offenkundig sind Strategien, die auf gesellschaftlichen und politischen Einfluss und die öffentliche Wahrnehmung der Marke abzielen („soft power“). Wirtschaftliche Interessen werden mittels Lobbyings der Politik oder des Platzierens von Interessenvertretern in Entscheidungsgremien verfolgt [35]. Vor der Veröffentlichung des WHO-Berichts zur gesunden Ernährung im Jahr 2003, in dem bereits damals ein Zuckerkonsum von maximal 10 Energieprozent empfohlen wurde, forderte die Zuckerindustrie zum Beispiel vom Kongress der Vereinigten Staaten, der WHO die finanziellen Mittel zu streichen [36]. Zu den Industrietaktiken gehören auch Versuche, wissenschaftliche Forschung zu beeinflussen, die Effektivität von staatlichen Regulierungen in Zweifel zu ziehen und stattdessen auf Selbstregulierung zu pochen, die Imagepflege, z. B. durch das Sponsern von Sport- und Kulturveranstaltungen oder Gesundheitskampagnen, sowie die ständige Betonung der persönlichen Verantwortung des Verbrauchers [37].

Aus diesen Beispielen wird deutlich, dass die Zuckerindustrie bei der Wahrnehmung ihrer Profitinteressen einen erheblichen Einfluss auf das Ernährungsverhalten und damit auf die Gesundheit der Bevölkerung ausübt. Diese Strategien müssen von der Gesundheitspolitik erkannt werden, um ihnen effektiv entgegenwirken zu können.

## Implikationen und Strategien für die Gesundheitspolitik

Die WHO-Richtlinie zum Zuckerkonsum wurde angesichts des wissenschaftlichen Konsenses über die kausale Rolle freier Zucker bei der Entstehung von Übergewicht und Zahnkaries entwickelt. Einzelne Staaten stehen nun vor der Herausforderung, Bedingungen zu schaffen, unter denen diese Empfehlungen erreicht werden können. Es besteht ein dringender Handlungsbedarf.

Ein Großteil der Gesundheitspolitik konzentriert sich nach wie vor auf den Versuch, den einzelnen Verbraucher oder Patienten durch Kenntnisvermittlung zu gesünderem Verhalten zu bewegen. Leider haben bisherige Evaluierungen gezeigt, dass derartige Interventionen bestenfalls kurzfristig erfolgreich sind [38] und soziale Ungleichheiten sogar verstärken können. Zum Beispiel zeigte die Evaluierung einer informationsbasierten Mundgesundheitskampagne für Schulkinder in Schottland, dass Verbesserungen bei Mundhygiene und Zahnfleischzustand nur bei Kindern in den weniger sozial benachteiligten Schulen erreicht wurden [39]. Das Problem einer alleinigen Fokussierung auf individuelle Verhaltensänderung besteht auch darin, dass die Schuld für eine schlechte Gesundheit auf den Einzelnen übertragen wird („victim blaming“; [40]). Für politische Entscheidungsträger mag das attraktiv sein, da eine derartige Sichtweise die Politik aus der Verantwortung entlässt. Die oben diskutierten sozialen und kommerziellen Einflüsse werden dabei aber nicht berücksichtigt. Das US-amerikanische Institute of Medicine hat es treffend formuliert: „Man kann nicht erwarten, dass Menschen ihr Verhalten einfach ändern, wenn sich so viele Kräfte im sozialen, kulturellen und materiellen Umfeld gegen solche Veränderungen verschworen haben“ [41].

Das bedeutet nicht, dass Gesundheitsaufklärung unwichtig ist. Aber leider hat in Deutschland die Zahl der Menschen, die sich im Umgang mit Gesundheits- und Präventionsempfehlungen überfordert fühlen, im Zeitverlauf zugenommen: Laut einer aktuellen Studie hatten 2 Drittel der Befragten eine geringe Gesundheitskompetenz, welche außerdem sozial ungleich verteilt war [42]. Hieraus wird klar, dass die Bevölkerung Unterstützung braucht und gesundes Verhalten leichter gemacht werden muss.

### Prinzipien und Konzepte der Gesundheitsförderung

#### Gemeinsamer Risikofaktorenansatz

Ein anerkanntes Prinzip der Mundgesundheitsförderung ist der *gemeinsame Risikofaktorenansatz* (Common Risk Factor Approach, CRFA; [43]). Der CRFA ist eine ganzheitliche Perspektive in Anerkennung der Tatsache, dass die wichtigsten Risikofaktoren sowohl Mund- als auch Allgemeinerkrankungen gemein sind. Neben Tabak- und Alkoholkonsum gehören dazu eine ungesunde, zuckerreiche Ernährung sowie die bereits beschriebenen sozialen und kommerziellen Gesundheitsdeterminanten. Die Implikation eines solchen Ansatzes ist die Kräftebündelung durch interdisziplinäre Zusammenarbeit, das heißt die Berücksichtigung von sowohl Mund- als auch Allgemeingesundheit in Empfehlungen und Präventionsprogrammen [25].

#### Hochrisikostrategie und Bevölkerungsstrategie

Geoffrey Rose hat in seinen Ausführungen zu Hochrisiko- und Bevölkerungsstrategien [44] gezeigt, dass bei häufigen chronischen Erkrankungen die Mehrzahl neuer Fälle (Inzidenz) bei denjenigen auftritt, die nicht zur Hochrisikogruppe gehören, weil „Hochrisikogruppe“ das extreme Ende der Verteilungskurve definiert. Hochrisikostrategien versuchen, diejenigen mit der größten Anfälligkeit für die jeweilige Krankheit durch einen Screeningprozess zu identifizieren, um ihnen individuelle Maßnahmen anzubieten, während Bevölkerungsstrategien auf die Ursachen der Krankheit abzielen. Eine erfolgreiche Hochrisikostrategie schneidet demnach den Hochrisikobereich der Verteilungskurve ab, eine erfolgreiche Bevölkerungsstrategie hingegen verschiebt die gesamte Kurve nach links. Bei der Hochrisikostrategie erhält der Einzelne eine Intervention aufgrund seines individuellen Krankheitsrisikos, allerdings setzt dies ein effektives Screening voraus, ist mit erheblichen Kosten verbunden und beseitigt oft nicht die Krankheitsursachen. Bevölkerungsstrategien können weit mehr neue Fälle verhindern als Hochrisikostrategien und daher hocheffektiv sein (Beispiele sind Maßnahmen zur Tabakkontrolle, Impfkampagnen oder die Sicherheitsgurtpflicht). Ein Mittelweg ist die Teilpopulationsstrategie, bei der sich Maßnahmen auf Bevölkerungsgruppen mit erhöhtem Krankheitsrisiko konzentrieren, ohne dass Einzelne ein Screening durchlaufen müssen. Alle 3 Strategien sollten sich auf sinnvolle Weise ergänzen [44]. Da sie mit weitaus größeren Gesundheitsgewinnen einhergehen, sollten Bevölkerungsstrategien angewendet werden, wann immer dies möglich und akzeptabel ist [45].

#### Upstream- und Downstream-Ansätze der Prävention

**Figure 3:**
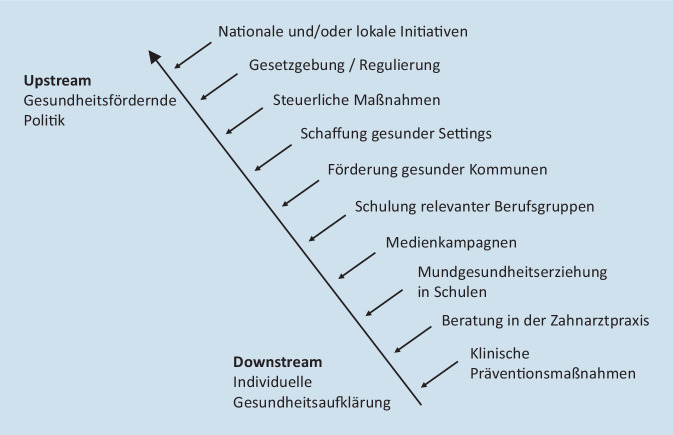

Die Upstream-Downstream-Metapher beschreibt die Idee, dass es effektiver ist, Krankheiten so weit wie möglich „upstream“, also ursächlich an ihrer Quelle, zu bekämpfen. Upstream-Ansätze basieren hauptsächlich auf Bevölkerungsstrategien. Upstream- und Downstream-Ansätze wurden im Kontext der Mundgesundheit vorgestellt [40]. Das Ziel ist eine sinnvolle Kombination von Upstream‑, Midstream- und Downstream-Interventionen. Abb. 3 zeigt Beispiele für die verschiedenen Ansätze, wobei Midstream-Strategien sich hauptsächlich auf lokal umsetzbare Maßnahmen in Zusammenarbeit mit u. a. öffentlichen Einrichtungen, Arbeitgebern und Einzelhandel beziehen [40].

### Upstream‑, Midstream- und Downstream-Ansätze zur Reduzierung des Zuckerkonsums

#### Upstream

Jahrzehntelange Erfahrungen mit der Tabakindustrie haben gelehrt, dass eine Kombination verschiedener Präventionsmaßnahmen notwendig ist und dass die wirksamsten Strategien auf ganzen Bevölkerungsansätzen und gesetzlichen Regulierungen beruhen [46]. Erfahrungen andernorts haben gezeigt, dass mit freiwilligen Ansätzen, wie sie vom Bundesministerium für Ernährung und Landwirtschaft (BMEL) bei der Zuckerreduktion favorisiert werden [47], ein substanzieller Fortschritt unwahrscheinlich ist, da die Ziele und Interessen der Industrie den Gesundheitsinteressen der Bevölkerung diametral entgegenstehen. So wurde zum Beispiel Großbritanniens „Responsibility Deal“ von Public-Health-Experten evaluiert und als gescheitert angesehen [48].

##### Zuckersteuer.

Die Besteuerung zuckerhaltiger Getränke in Abhängigkeit von der enthaltenen Zuckermenge ist eine der wirksamsten Maßnahmen zur Reduzierung des Zuckerkonsums und wurde weltweit bereits in mehr als 40 Ländern eingeführt [49]. Die Effektivität von Preiserhöhungen ab 20 % wurde in verschiedenen Evaluations- und Simulationsstudien gezeigt [49, 50]. So reagierte z. B. im Vereinigten Königreich die Softdrinkindustrie auf die Ankündigung der 2018 eingeführten Zuckersteuer mit der Reduzierung des Zuckeranteils in ihren Getränken um durchschnittlich 38 %, der Marktanteil von Getränken mit mehr als 5 g Zucker pro 100 ml sank um etwa 34 % [51].

In Deutschland wird die Einführung einer „Zucker- oder Soft-Drink-Steuer“ von Ärzten, Zahnärzten und Wissenschaftlern klar befürwortet (siehe Infobox 1; [52, 53]). Modellrechnungen zeigen, dass die Einführung einer Zuckersteuer in Deutschland zu moderaten Rückgängen sowohl von Karies als auch Übergewicht führen würde. Eine solche Steuer wäre regressiv, d. h., Verbraucher mit geringerem Einkommen wären stärker von den Preissteigerungen betroffen, da sie die besteuerten Produkte im Durchschnitt häufiger konsumieren. Auf der anderen Seite ginge sie aber für genau diese Konsumenten auch mit den größten Gesundheitseffekten einher und könnte somit zur Verringerung sozialer Ungleichheiten beitragen [54, 55]. Die Regressivität der Steuer sollte durch Subventionierung gesünderer Lebensmittel ausgeglichen werden.

##### Werbeverbote.

Die Marketingstrategien der Zuckerindustrie bedürfen wesentlich strikterer Kontrolle als momentan der Fall. Die WHO empfiehlt z. B. seit 2017 ein komplettes Werbeverbot für ungesunde Produkte, die an Kleinkinder gerichtet sind [56]. Aktuell gibt es in Deutschland leider keine verbindlichen Einschränkungen für die Vermarktung ungesunder Lebensmittel an Kinder [57]. Der diesbezügliche Ansatz des BMEL, lediglich Zuckerzusätze in Tees für Babys und Kleinkinder zu verbieten [58], greift viel zu kurz. Eine weitreichendere Regulierung des Marketings von zuckerreichen Lebensmitteln an Kinder, wie von WHO [59] und Robert Koch-Institut [60] empfohlen, ist dringend geboten.

##### Nährwertkennzeichnung.

Eine weitere wichtige Upstream-Strategie ist eine obligatorische und leicht verständliche Nährwertkennzeichnung, um Verbrauchern beim Treffen gesünderer Kaufentscheidungen zu helfen. In Deutschland trat im November 2020 die Verordnung zur Einführung des Nutri-Scores (System zur Nährwertkennzeichnung von Lebensmitteln) in Kraft. Dies ist begrüßenswert, ob allerdings der Nutri-Score den gewünschten Effekt haben wird, bleibt fraglich. Denn die Kennzeichnung erfolgt auf freiwilliger Basis, da das geltende EU-Recht eine verpflichtende nationale Anwendung nicht ermöglicht. Innerhalb der EU-Ratspräsidentschaft 2020 setzte sich Deutschland deshalb für eine EU-weit einheitlich erweiterte Nährwertkennzeichnung ein. Derartige Bemühungen sollten von zahnärztlichen Berufsvertretern unterstützt werden.

##### Präventionsgesetz.

Im Jahr 2015 wurde in Deutschland das Präventionsgesetz verabschiedet, welches gesundheitsförderliche Lebenswelten und Verhaltensweisen unterstützen sowie soziale Ungleichheiten von Gesundheitschancen verringern soll. Allerdings findet die Bedeutung freier Zucker für die Mundgesundheit im Gesetz keine Erwähnung. Hier bestehen Chancen für eine Zusammenarbeit im Sinne des gemeinsamen Risikofaktorenansatzes. Bei der geplanten Novellierung des Gesetzes sollten die Mundgesundheitsförderung unbedingt einbezogen und bereits vorhandene Strukturen genutzt werden, wie z. B. die zahnmedizinische Gruppenprophylaxe nach § 21 SGB V (Sozialgesetzbuch, Fünftes Buch).

#### Midstream

Zu den Midstream-Strategien gehört die Schaffung gesundheitsförderlicher Lebenswelten, z. B. in Kitas, Schulen, Betrieben und Krankenhäusern. Gesundheitsfördernde Settings können auch jene Zielgruppen erreichen, die von individuellen verhaltensbezogenen Präventionsangeboten wenig profitieren. Die Deutsche Gesellschaft für Ernährung (DGE) hat für verschiedene Lebenswelten Qualitätsstandards entwickelt, die als Mindeststandards verbindlich gemacht werden sollten [61].

Kitas und Schulen bilden ein ideales Umfeld für die Umsetzung einer (mund‑)gesunden Ernährung. Sie können außerdem gesundheitliche Chancengleichheit fördern, vor allem für Kinder aus Familien, in welchen Ernährungsrituale und entsprechende Strukturen fehlen. Eine gesunde, zuckerarme Ernährung zur Prävention von Karies und Übergewicht sollte durch verbindliche, auf Empfehlungen der DGE basierende Richtlinien für Kita- und Schulessen gewährleistet werden. Die Zusammenarbeit mit den pädagogischen Fachkräften, in deren Aufgabenbereich die Gesundheitsbildung in Kita und Schule fällt, ist dabei wesentlich. Ein Beispiel für ein erfolgreiches Kitapräventionsprogramm ist das inzwischen von verschiedenen Landesarbeitsgemeinschaften für Jugendzahnpflege übernommene, ursprünglich Brandenburger Gruppenprophylaxe-Konzept „Kita mit Biss“ – ein Aufklärungs- und Ernährungsprogramm, das einen mundgesundheitsförderlichen Kitaalltag etablieren soll [62].

Auch im Betrieb können gesundheitliche Rahmenbedingungen für beschäftigte Erwachsene gezielt beeinflusst werden. Auch hier geht es darum, die Verhältnisse am Arbeitsplatz gesundheitsförderlich zu gestalten. Konkret auf die Ernährung bezogen, sollte das Essen am Arbeitsplatz und in der Kantine abwechslungsreich, vollwertig sowie zucker-, fett- und salzarm zubereitet werden. Auf Fertigprodukte sollte verzichtet werden, um versteckten Zuckerverzehr zu vermeiden.

Weitere Möglichkeiten der Integration von Botschaften zur Mundgesundheitsprävention bietet der Nationale Aktionsplan „IN FORM – Deutschlands Initiative für gesunde Ernährung und mehr Bewegung“ [63], der die Prävention von Fehlernährung, Bewegungsmangel, Übergewicht und damit zusammenhängenden Krankheiten zum Ziel hat. Zu den Maßnahmen gehört z. B. die Einrichtung von „Vernetzungsstellen Kita- und Schulverpflegung“ in den Bundesländern zur Förderung von Ernährungswissen in Schulen, welche von der Einbeziehung mundgesundheitlicher Expertise profitieren könnten.

Weitere Midstream-Ansätze sind die Entfernung von Süßwaren aus dem Kassenbereich von Supermärkten, Tankstellen und anderen Geschäften (sogenannte Quengelware) sowie die Regulierung von Preispromotionen für stark zuckerhaltige Produkte [64, 65].

#### Downstream

Zahnärztinnen und Zahnärzte sind eine von der Bevölkerung regelmäßig frequentierte und häufig kontrollorientiert in Anspruch genommene Arztgruppe. Deshalb sind die zahnärztlichen Praxen besonders für gesundheitliche Aufklärung und für eine Informationsvermittlung zur gesundheitsförderlichen Verhaltensänderung geeignet [66]. Die Ernährungsberatung durch Zahnärzte und Zahnärztinnen sollte verstärkt werden. Eine solche Beratung muss dabei dem aktuellen Stand der Ernährungsforschung entsprechen und auf evidenzbasierten Methoden beruhen. Um den (versteckten) Zuckerverzehr bei Säuglingen und Kindern zu minimieren, ist außerdem die interdisziplinäre Zusammenarbeit mit Pädiatern, Gynäkologen und Hebammen (Schwangerenberatung) wichtig.

## Fazit

Zucker spielt eine ursächliche Rolle bei der Entwicklung von Karies und Übergewicht und ist daher eine wichtige kommerzielle Gesundheitsdeterminante sowohl für die Mund- als auch die Allgemeingesundheit. Strategien zur Reduzierung des Zuckerkonsums sollten daher disziplinübergreifend und in enger Zusammenarbeit mit anderen Gesundheitsberufen und Public-Health-Akteuren verfolgt werden, um Fachlichkeit und gesundheitspolitischen Einfluss zu bündeln. Prävention ist dann am effektivsten, wenn sie der Rolle sozialer, materieller und kommerzieller Einflüsse Rechnung trägt. Dazu bedarf es einer umfassenden Gesundheitspolitik, welche durch eine sinnvolle Kombination von Upstream‑, Midstream- und Downstream-Strategien Lebenswelten schafft, die gesundes Verhalten fördern und erleichtern. Ganz konkret können die verbindliche Einführung einer Ernährungsampel, die Erhebung einer Zuckersteuer auf zuckerhaltige Getränke, Werbeverbote sowie verbesserte Ernährungsprogramme in den Settings Kita und Schule einen entscheidenden Beitrag zur Zuckerreduktion und damit zur Verbesserung der Mund- und Allgemeingesundheit leisten. Die zahnmedizinische Gemeinschaft aus Wissenschaftlern, Praktikern und berufspolitisch Engagierten ist in einer idealen Position, um dabei ihre Expertise einzubringen. Wir möchten eine entsprechende Vernetzung anregen und unterstreichen die Notwendigkeit der Evaluierung existierender Präventionsprogramme sowie weiterer Studien insbesondere zur Effektivität von Midstream- und Downstream-Ansätzen.

### Infobox 1 Position der Bundeszahnärztekammer zum Thema gesunde Ernährung, wortwörtlich zitiert aus [53]

1. Der Verbraucher hat ein Recht auf eine verständliche Lebensmittelkennzeichnung, insbesondere im Hinblick auf die Menge zuckerhaltiger Nahrungsbestandteile und ungünstiger Fettsäuren.
2. Insbesondere Lebensmittel für Kleinkinder sollten deutlich zuckerreduziert, mit einer klaren Lebensmittelkennzeichnung (speziell auf Zucker) versehen sein und deutlichen Beschränkungen für die Lebensmittelwerbung unterliegen.
3. Die Einführung von Sonderabgaben für stark zucker- und/oder säurehaltige sog. Softdrinks ist eine sinnvolle Maßnahme, wie das Beispiel anderer Länder zeigt.
4. Es braucht verbindliche Standards für eine ausgewogene, gesunde Schul- und Kitaverpflegung.
5. Die Verhältnis- und Verhaltensprävention im Bereich der Ernährung sollte durch Maßnahmen der Präventionsgesetzgebung unterstützt werden.
6. Die Gruppenprophylaxe (§ 21 SGB V) sollte auch zur Vermittlung einheitlicher Standards von Ernährungsempfehlungen genutzt werden.

## Footnotes

1 „Frisch“ bedeutet hier ganzes Obst und Gemüse mit intakter Zellstruktur, schließt also Fruchtsäfte oder getrocknetes Obst nicht mit ein.

2 Nachdrückliche Empfehlungen werden von der WHO ausgesprochen, wenn die Forschungslage zeigt, dass bei Einhaltung der Empfehlung die erwünschten Effekte überwiegen und die Empfehlung in den meisten Situationen umsetzbar ist. Ergänzende Empfehlungen werden dann ausgesprochen, wenn über die Balance zwischen Vor- und Nachteilen der Umsetzung weniger Sicherheit besteht [14].
